# Trends of reported foodborne diseases at the Ridge Hospital, Accra, Ghana: a retrospective review of routine data from 2009-2013

**DOI:** 10.1186/s12879-016-1472-8

**Published:** 2016-03-24

**Authors:** Benjamin Osei-Tutu, Francis Anto

**Affiliations:** School of Public Health, University of Ghana, Legon, Accra Ghana; Food and Drugs Authority Accra, Accra, Ghana

**Keywords:** Foodborne diseases, Cholera, Fever, Gastroenteritis, Viral hepatitis, Dysentery, Ridge Hospital

## Abstract

**Background:**

There are over 250 foodborne diseases and are of growing public health concern worldwide. The distribution of these diseases varies from one locality to the other. Foodborne diseases come about as a result of ingestion of food contaminated with microorganisms or chemicals. The most common clinical presentation of foodborne disease takes the form of gastrointestinal symptoms; although other systems of the body can also be affected and represents a considerable burden of disability as well as mortality. The current study was carried out with the aim of describing the trends and patterns of foodborne diseases reported at the Ridge Hospital in Accra, Ghana to serve as the first step towards understanding the profile of foodborne diseases in Accra. The study could then serve as a guide in the establishment of a sentinel site or surveillance system for foodborne diseases.

**Methods:**

A retrospective review of routine data kept on patients who visited the Ridge Hospital from January 2009 to December 2013 was conducted to describe the trends and patterns of foodborne diseases reported at the facility. All available health records were reviewed and data on foodborne diseases extracted and analysed by age group, sex, season and geographical location within the catchment area of the hospital.

**Results:**

The review showed significant variation in the annual reported cases of foodborne diseases [2009 = 11.5 % (118/1058); 2010 = 2.30 % (22/956); 2011 = 17.45 % (608/3485); 2012 = 7.98 % (498/6315) and 2013 = 2.56 % (345/13458)] *p* < 0.05. Significant seasonal variations were also observed [early dry season = 10.2 % (322/3142); late dry season = 24.4 % (909/3728); early wet season = 4.3 % (107/2494); late wet season = 6.3 % (256/4094). There were monthly variations also during the period (*p* < 0.001) except for the year 2010 (*p* = 0.428). The highest prevalence was reported during the late dry season (February–April). The most affected age group was those aged between 15 and 34 years who had significantly more infections in 2012 and 2013 than the other age groups (*p* < 0.001). Overall many more males than females reported of food borne diseases (*p* < 0.001).

**Conclusion:**

The commonly reported foodborne diseases at the Ridge Hospital were: typhoid fever, dysentery, cholera and viral hepatitis. These diseases were found to be very seasonal with peaks at the onset of the rainy season.

**Electronic supplementary material:**

The online version of this article (doi:10.1186/s12879-016-1472-8) contains supplementary material, which is available to authorized users.

## Background

Foodborne diseases (FBDs) are a major public health concern in both developed and developing countries, as it comprise a broad spectrum of diseases and accounts for a significant proportion of morbidities and mortalities worldwide [1]. There are over 250 FBDs and the distribution of these diseases varies from one locality to the other [2–4]. Even though the causative agents of most cases of foodborne diseases are unknown, bacteria and viruses are the most likely causative agents [5, 6].

Foodborne diseases result from the consumption of food contaminated with pathogens such as bacteria, viruses, parasites or with poisonous chemicals or bio-toxins [7, 8]. Most foodborne disease cases are mild and self-limiting however severe cases can occur in high risk groups, such as infants, young children, the elderly and the immunocompromised persons, resulting in high mortality and morbidity among this group [9]. Gastroenteritis, which is a syndrome of diarrhea and/or vomiting, is a major cause of morbidity and mortality in children, especially in developing countries. Most cases of gastroenteritis are caused by foodborne pathogens [10–12]. However, it is important to note that diarrhea and vomiting are non-specific symptoms that may be caused by other diseases.

The spectrum of foodborne diseases changes with demographic and epidemiologic changes in the population. Several factors contribute to the incidence of foodborne diseases in a particular locality. These include host factors (age and sex) and environmental factors (season and geographical location). Some predisposing factors that affect the rapid transmission of foodborne diseases in a locality are poor personal hygiene, poor sanitation, attitudes toward food hygiene and overcrowding. Understanding the epidemiology of FBDs in a particular locality requires the establishment of sentinel sites or a surveillance system. However, to establish a surveillance system one needs to know the profile of possible FBDs and the trends and pattern in that locality in order to know the ones to monitor. Ghana is yet to have sentinel sites or any documented surveillance system for FBDs. This study was therefore aimed at describing the trends and patterns of foodborne diseases reported at the Regional Hospital of the national capital, Ridge Hospital, between 2009 and 2013 as the first step towards understanding the profile of FBDs in Accra. Diseases captured in this review included those normally acquired via the faeco-oral route through contaminated food, contaminated drinking water or beverage. The study could then serve as a guide in the establishment of a sentinel site or surveillance system for FBD.

## Methods

### Study site

**Fig. 1:**
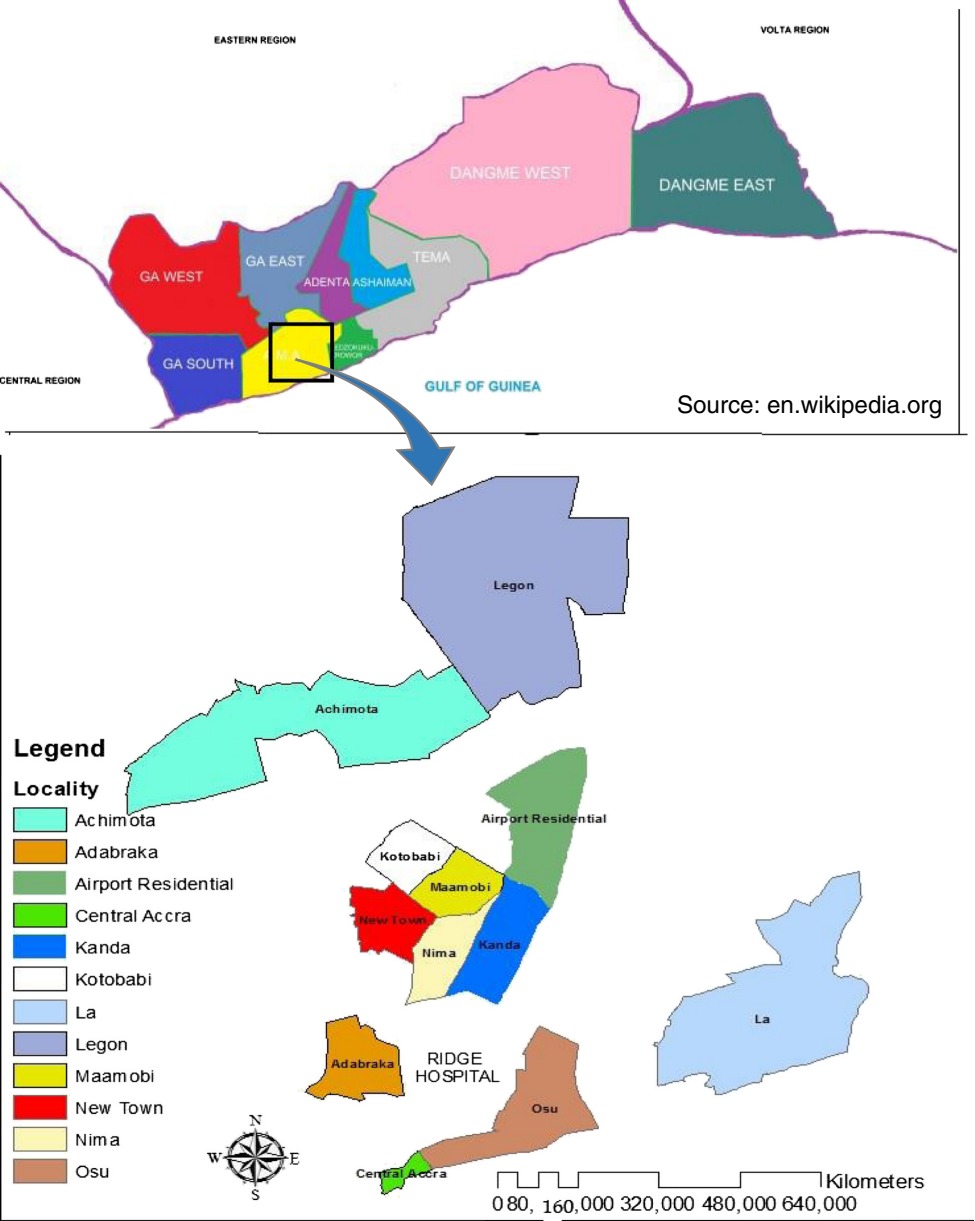
Map of Greater Accra Region showing the localities within the catchment areas of the Ridge Hospital

The study was carried out at the Ridge Hospital located within the Osu Klottey sub Metropolis of the Accra Metropolitan Area. It is the Regional Hospital for the Greater Accra Region. The immediate catchment area of the hospital includes the following communities: Nima, Maamobi, Kanda, Accra New Town, Kotobabi, Osu, La, Adabraka, Airport residential, Legon, Achimota and Central Accra (Fig. 1). The hospital has a bed capacity of 194 beds and staff strength of 400. The average daily out-patient (OPD) attendance and admissions are 388 and 43 respectively. The facility provides 16 out-patient, nine in-patient and 10 specialized services. Some of the out-patient services include general OPD, accidents and emergency services, laboratory and public health. Some of the in-patients services include surgical, post natal and neonatal intensive care. The hospital also has a poisoning center that handles all poisoning cases including food poisoning.

### Study population and design

The study involved a retrospective review of routine data kept on patients who visited the Ridge Hospital. The study population was made up of all patients who visited the hospital for treatment, during the period January 2009 and December 2013. Out Patient and Inpatient Departments registers were reviewed to obtain clinical diagnosis of all cases during the period. This was done by sequentially reviewing all the records of patients, beginning from January 2009 to December 2013. Details such as age, sex, date of visit and place of residence of patients were extracted from the registers. Laboratory records were also reviewed to obtain the number of confirmed cases.

### Data collection methods & tools

The data collection tool was designed as a line listing form with rows and columns. The columns had the following headings; record number, clinical diagnosis, date of visit, sex of patients, age and place of residence. Each row contained information on a patient diagnosed with FBDs (Additional file 1).

Consulting room and general ward registers had columns with headings such as age, sex, place of residence and diagnosis (Additional files 2 & 3). Each row in the register represented the record of a patient treated in the hospital. These registers were reviewed to obtain the principal diagnosis of out-patients. Principal diagnosis that falls in a particular category of waterborne/foodborne disease of public health concern were extracted. Classification of cases into the various categories of waterborne/foodborne diseases was done using the International Classification of Diseases (ICD-10) [13]. Most of the diagnoses were based on clinical assessment with only 8.0 % being laboratory confirmed. In Ghana, clinicians routinely use the Integrated Disease Surveillance and Response Ghana (IDSR) [14]. Details such as age, sex, date of visit and place of residence of patient diagnosed with waterborne/foodborne diseases were also obtained from these registers using the data abstraction form.

The demographic characteristics of the various places of residence such as the population density, education level, employment status and geographical coordinates were obtained from the Ghana Statistical Service. To obtain data on population density, education level, employment status and geographical coordinates, the place of residence was linked with the demographic data of the town in which the patient lived.

### Data processing & analysis

Measurements were done using aggregated measures (prevalence) for the FBDs and demographic measures for the place of residence. Thus analysis was done using individual information of FBDs and other variables but group information for place of residence. The data was coded and refined using Epidata 3.1 software. After checking for consistency and completeness of data, the records were then exported into Stata Version 12 and analyzed statistically using both Stata Version 12 and Microsoft Excel 2013. Baseline characteristics of the population (records with complete data on patients treated) were explored using simple descriptive method such as frequency distribution. Prevalence of the types of waterborne/foodborne diseases was expressed as a percentage of the total number of patients treated for the period. Prevalence of FBDs was compared among the different localities (place of residence) during the same period and over time. In comparing the various prevalence, a chi square test for trend was used to find significant differences (*p* <0.05) among the different groups.

ArcGIS 10.1 software was used to do spatial analysis of the data. Digital boundary files for the Enumerated Areas (EAs) as defined by the Ghana Statistical Service were created to obtain geographical maps of the localities within catchment areas of the hospital. This was then used to summarize the demographic data for each of the locality in the catchment area of the hospital.

### Ethical issues

Ethical clearance was obtained from the Ghana Health Service Ethical Review Committee (ID NO: GHS-ERC: 39/04/14). Permission was also obtained from the Medical Superintendent of the Ridge Hospital. The study was conducted in accordance with Good Clinical Practice, Declaration of Helsinki and applicable local regulations. This study was wholly a review of secondary data and so obtaining written informed consent was not a requirement after ethical approval from the Ghana Health Service and permission from the management of the health facility.

## Results

### Background characteristics of study participants

In all, 46,432 patient records covering the period 2009–2013 were reviewed for this study. Out of this number, 25,272 (54.4 %) had complete data (age, sex, place of residence, clinical diagnosis, date of attendance, indication of laboratory test) and therefore used in this analysis. There was a progressive increase in hospital attendance over the period except for the year 2010, the only year that there was no reported case of cholera (2009: 1,058; 2010: 956; 2011: 3,485; 2012: 6,315 and 2013: 13,458). The number of records reviewed per month for the 5 years was between 1,732 and 2,511. The participants were made up of 10,516 (41.6 %) males and 14,756 (58.4 %) females aged 0 – 83years (mean = 29.4 years; SD ±15.4), mainly resident within the catchment area of the health facility. A total of 1,591 (6.3 %) patients were diagnosed of food borne diseases. This was made up of 870 (54.7 %) males and 721 (45.3 %) females (Table 1). The mean ages of the patients were similar over the period (2009: 28years, SD ±18.6; 2010: 31years, SD ±14.6; 2011: 30 years, SD ±14.1; 2012: 30 years, SD ±15.1; 2013: 28years, SD ±18.6).

**Table 1:** Prevalence of reported Foodborne Diseases by demographic characteristics of study population: 2009–2013

Characteristics of patients	2009 (*N* = 118)		2010 (*N* = 22)		2011 (*N* = 608)		2012 (*N* = 498)		2013 (*N* = 345)	
	n	(%)	n	(%)	n	(%)	n	(%)	n	(%)
Sex	X^2^ = 0.57		X^2^ = 4.05		X^2^ = 11.32		X^2^ = 38.56		X^2^ = 5.36	
	*P* = 0.901		*P* = 0.256		*P* = 0.010		*P* < 0.001		*P* = 0.373	
Male	55	(46.6)	12	(54.5)	364	(59.9)	285	(57.2)	154	(44.6)
Female	63	(53.4)	10	(45.5)	244	(40.1)	213	(42.8)	191	(55.4)
Age (years)	X^2^ = 22.34		X^2^ = 14.09		X^2^ = 20.78		X^2^ = 69.30		X^2^ = 61.09	
	*P* = 0.099		*P* = 0.519		*P* = 0.144		*P* < 0.001		*P* < 0.001	
<5	11	(9.3)	1	(4.5)	14	( 6.3)	16	(3.2)	47	(13.6)
5-14	19	(16.1)	2	(9.1)	42	(15.2)	28	(5.6)	35	(10.1)
15-24	28	(23.7)	4	(18.2)	191	(53.8)	174	(34.9)	84	(24.3)
25-34	20	(17.0)	4	(18.2)	171	(16.0)	130	(26.1)	71	(20.6)
35-44	15	(13.6)	8	(36.4)	96	(13.7)	72	(14.5)	44	(12.8)
>45	24	(20.3)	3	(13.6)	94	(10.9)	78	(15.7)	64	(18.6)
Place of residence	X^2^ = 52.22		X^2^ = 15.09		X^2^ = 34.01		X^2^ = 57.64		X^2^ = 46.53	
	*P* = 0.039		*P* = 0.655		*P* = 0.419		*P* = 0.562		*P* = 0.899	
Nima	7	(5.9)	3	(13.6)	93	(15.3)	72	(14.4)	39	(11.3)
Maamobi	4	(3.4)	5	(22.7)	14	(2.3)	37	(7.4)	17	(4.9)
Kanda	1	(0.8)	0	(0)	12	(2.0)	17	(3.4)	20	(5.8)
Accra Newtown	6	(5.1)	2	(9.1)	11	(1.8)	29	(5.8)	19	(5.5)
Kotobabi	1	(0.8)	0	(0)	13	(2.1)	12	(2.4)	2	(0.6)
Osu	14	(11.9)	2	(9.1)	19	(3.1)	15	(3.0)	14	(4.1)
La	7	(5.9)	0	(0)	12	(2.0)	10	(2.0)	8	(2.3)
Adabraka	28	(23.7)	2	(9.1)	229	(37.7)	88	(17.7)	78	(22.6)
Airport residential	2	(1.7)	0	(0)	0	(0)	10	(2.0)	4	(1.2)
Legon	2	(1.7)	0	(0)	5	(0.8)	14	(2.8)	3	(0.9)
Achimota	4	(3.4)	0	(0)	6	(1.0)	7	(1.4)	10	(2.9)
Central Accra.	9	(7.6)	3	(13.6)	10	(1.6)	26	(5.2)	25	(7.2)
Others	33	(28.0)	5	(22.7)	184	(30.3)	161	(32.3)	106	(30.7)

### Reported food borne diseases

Four main clinically diagnosed food borne diseases were reported during the period 2009 to 2013. These were: cholera (59.8 %), typhoid fever (16.5 %), dysentery [shigellosis] (2.6 %), and viral hepatitis (1.6 %). Only 8.0 % (127/1,591) of the cases were however confirmed through laboratory analysis (gastroenteritis: 64; cholera: 61 and hepatitis A: 2). In the year 2009, only 4.2 %, (5/118) of the clinical cases were laboratory confirmed. The highest proportion of laboratory confirmed cases was recorded in 2010 when 6 out of 22 (27.3 %) cases were confirmed. In 2013, no clinical case was confirmed through laboratory analysis.

Significant differences in prevalence of reported cases of foodborne diseases were observed over the 5-year period. The highest prevalence of reported cases was in 2011 (17.4 %, 608/3485) while the lowest was recorded in 2010 (2.3 %, 22/956). Significant monthly variations in the number of reported food borne diseases was also observed (*p* < 0.001) throughout the period except for the year 2010 (*p* = 0.428). Significant seasonal variations were also observed, with the highest prevalence (24.4 %, 909/3728) occurring during the late dry season (February –April). Reports for the other seasons were: early dry season = 10.2 % (322/3142), late dry season = 24.4 % (909/3728), early wet season = 4.3 % (107/2494) and late wet season = 6.3 % (256/4094)]. Monthly variations were also observed (*p* < 0.001) except for the year 2010 (*p* = 0.428). The most affected age group was those aged between 15 and 34 years who had significantly more infections in 2012 and 2013 than the other age groups (*p* < 0.001). Overall, many more males than females reported of food borne diseases (*p* < 0.001) [Table 1].

**Fig. 2:**
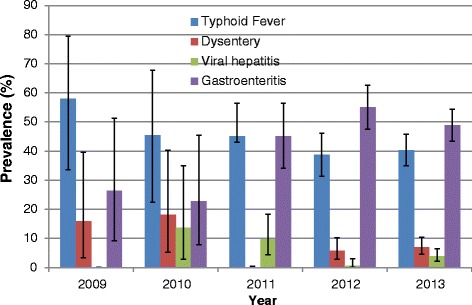
Annual Reported Non-Cholera Foodborne Diseases at the Ridge Hospital, Accra: 2009–2013

Typhoid fever was the only specific food borne disease that was consistently reported throughout the five years. The prevalence of this infection appears to be coming down gradually whilst the level of reported gastroenteritis is increasing over the period, though the observed difference was not statistically significant (Fig. 2). The review revealed that, there were outbreaks of cholera in 2009, 2011 and 2012 with the highest number of cases (526) being reported in 2011, with no reported case in 2010 (Table 2).

**Table 2:** Reported Foodborne Diseases at the Ridge Hospital: 2009–2013

Type of FBDs reported	2009		2010		2011		2012		2013		Total	
	n	(%)	n	(%)	n	(%)	n	(%)	n	(%)	n	%
Typhoid Fever	11	(9.3)	10	(45.5)	37	(6.1)	68	(13.7)	137	(39.7)	263	(16.5)
Dysentery	3	(2.5)	4	(18.2)	0	(0)	10	(2.0)	24	(7.0)	41	(2.6)
Cholera	99	(83.9)	0	(0)	526	(86.5)	322	(80.3)	5	(1.4)	952	(59.8)
Viral Hepatitis	0	(0)	3	(13.6)	8	(1.3)	1	(0.2)	13	(3.8)	25	(1.6)
Gastroenteritis ^a^	5	(4.2)	5	(22.7)	37	(6.1)	94	(18.9)	165	(47.8)	306	(19.2)
Others	0	(0)	0	(0)	0	(0)	3	(0.6)	1	(0.3)	4	(0.3)
Total	118	(100)	22	(100)	608	(100)	498	(100)	345	100	1591	(100)

^a^about 71 % could be attributed to etiologic agents of FBD [19]

### Seasonality of the food borne diseases

**Fig. 3:**
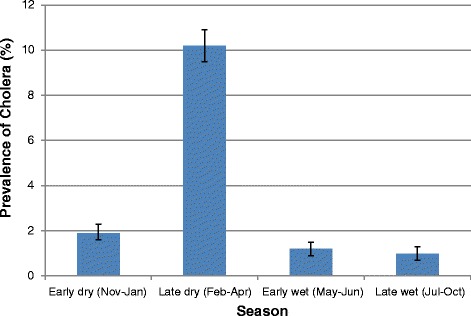
Seasonal Prevalence of Reported Cholera at the Ridge Hospital, Accra: 2009–2013

**Fig. 4:**
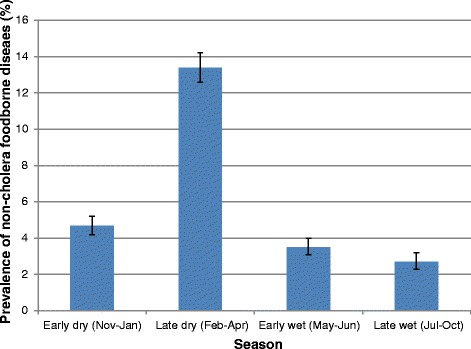
Seasonal Prevalence of Reported Non-Cholera foodborne diseases at the Ridge Hospital, Accra: 2009–2013

The records revealed that cholera was very seasonal with a peak during the months of February to April, which coincides with the end of the dry season and start of the rainy season. During the other months of the year, (November to January, May to June and July to October), lower prevalences (<2.0 %) were reported (Fig. 3). Combining all the reported non-cholera foodborne diseases also shows a high level of seasonality with a peak during the months of February to April, with a pattern similar to that of cholera (Fig. 4).

### Geographical pattern of Food borne disease

Among the localities within the catchment area of the Ridge Hospital, Nima has the highest population density (705 persons per square kilometer) and percentage illiteracy (17.04). Percentage unemployed was high among the population in Legon (77.72). The lowest population density (25 persons per square kilometer) was recorded in Airport residential area and the lowest percentage illiteracy (2.96) was recorded in Legon. The lowest percentage unemployed (30.35) was recorded in Central Accra [15].

Prevalence of foodborne diseases reported from the various localities within the catchment area of the hospital was similar. High prevalence of foodborne diseases was recorded from communities with low percentage illiteracy and population density as well as those with high percentage illiteracy and population density. The highest prevalence of foodborne diseases was recorded among the patients from Nima (9.2 %; 214/2336) and the lowest was recorded among the patients from Kanda (4.2 %; 50/1200). No apparent relationship was therefore found between the prevalence of foodborne diseases and illiteracy rate or population density.

## Discussion

The current study was undertaken to describe the trends and pattern of diseases related to water and foodborne pathogens reported during the period 2009 to 2013 at the Ridge Hospital in Accra, Ghana and to provide a baseline that could serve as a guide in the establishment of sentinel sites or a surveillance system for diseases in the country. Analysis of the hospital based data revealed that cholera, typhoid fever, viral hepatitis and dysentery (shigellosis) were the most commonly reported water and foodborne diseases at the health facility during the period. These diseases were all found to be very seasonal with a peak during the months of February to April, which coincides with the end of the dry season and start of the rainy season.

A similar analysis of hospital data in Ibadan City, Nigeria by Oguntoke , Aboderin and Bankole [16] revealed typhoid fever, bacillary dysentery and cholera as the most commonly reported waterborne/foodborne diseases at the health facilities studied. They also observed marked seasonality in the prevalence of the cases. The occurrence of these diseases in our cities and towns has been attributed largely to unsafe water, inadequate sanitation and poor hygiene among the inhabitants. The high prevalence of cholera in the current study indicates that *Vibrio cholerae* is the main etiologic agent of waterborne/foodborne illnesses of public health concern within the catchment area of the Ridge Hospital in Accra.

Cholera is now endemic with cyclical epidemics in the major cities and towns in Ghana. Outbreaks are now predictable starting with the onset of rains. The onset of the rains, results in contamination of unprotected water source with latrine overflow and sewage. Outbreaks could occur when people drink from such water source or when such water comes into contact with food or food contact surfaces [17, 18]. Attempts at controlling the disease over the years have been unsuccessful because the needed long term approaches, such as potable water supply, proper disposal of solid waste etc. have not been implemented [19].

An earlier meta-analysis of publications on microbiological food safety in Ghana by Saba and Gonzalez-Zorn [20] reported *Enterobacter* spp., *Citrobacter* spp., *Klebsiella* spp. and *Escherichia* spp. as the predominant bacteria in Ghanaian foods. Using clinical symptoms alone for the diagnosis of waterborne/foodborne diseases in the current study could not establish the specific pathogenic agents responsible for the illnesses resulting in many of the cases being grouped together and classified as gastroenteritis by the clinicians. This may account for the yearly recording of gastroenteritis in the current study as only 8 % of the clinical diagnosis was confirmed through laboratory investigations. In a study conducted in the Netherlands, foodborne pathogens explained 71 % of the cases classified as gastroenteritis by general practitioners [21].

The seemingly steady trend of typhoid fever over the five year period (average of 1 %) could be due to a combination of factors: poor water and food hygiene, drug resistance and lack of health care infrastructure. Poor food hygiene practices in homes and the food service industry result in food being contaminated with *Salmonella typhi* [22, 23]*. S. typhi* is however becoming resistant to the commonly used antimicrobials for the treatment of typhoid fever in sub-Saharan Africa; including Nalidixic acid, chloramphenicol, ciprofloxacin and ceftriaxone. This, coupled with the lack of laboratories equipped with modern diagnostic tools to accurately detect H and O antibody titre levels in patients, complicate the management of typhoid fever in sub-Saharan Africa [24, 25].

The yearly prevalence of dysentery (shigellosis) among patients visiting the Ridge Hospital was generally low (<1 %) throughout the period, with the highest prevalence occurring during the late dry season (February – April). The prevalence of waterborne/foodborne diseases among patients visiting the hospital was relatively higher among the 15–24 years age group. This age group consists mainly of people in secondary schools and tertiary institutions and mostly rely on street vended foods or foods from restaurants. Street vended foods and foods from restaurants, however have been implicated in a number of studies as sources of foodborne pathogens. An earlier study on street vended foods in Accra found feacal coliform (1.0 × 10^2^ – 1.9 × 10^5^ cfu/ml) in 85 % of water sources used in preparing food in some local restaurants in Accra [26].

Interestingly, the under 5 year olds had relatively few cases compared to the other age groups. This may be attributable to the case definition of cholera, which excludes <5 years olds and also the availability of a children’s hospital, the Princess Marie Louise Children’s Hospital in Accra not far from the Ridge Hospital where the current study was undertaken.

## Conclusion

A cross-sectional descriptive study involving the review of health facility data was undertaken with the aim of providing baseline data that could serve as a guide in the establishment of a surveillance system for monitoring trends in water and foodborne diseases in Ghana. Analysis of the data revealed that the commonly reported waterborne/foodborne diseases at the Ridge Hospital were: typhoid fever, dysentery, cholera and viral hepatitis. These diseases were found to be very seasonal with peaks at the onset of the rainy season.

### Limitations

The unavailability of some consulting room registers and the incompleteness of data in most of the registers was a limitation in this study. This may have contributed to low reported cases of waterborne/foodborne diseases for some of the months. Another limitation was the absence of laboratory confirmation for most of the diagnoses. Hence diagnoses were based on the judgment of the clinician. This could lead to misclassification of diseases. The number of reported waterborne/foodborne diseases at the Ridge Hospital may only be a fraction of cases that actually occurred in the study area as some cases may reported to other health facilities in the municipality.

## Additional files

Additional file 1: Data abstraction form. (DOC 66 kb) Additional file 2: Consulting Room Register. (PDF 122 kb) Additional file 3: General Ward Register. (PDF 514 kb)
