# Supplementary material for: Trends of reported foodborne diseases at the Ridge Hospital, Accra, Ghana: a retrospective review of routine data from 2009-2013
Source: BMC Infect Dis. 2016 Mar 24;16:139. doi: 10.1186/s12879-016-1472-8 (PMC4807551; doi:10.1186/s12879-016-1472-8)
Supplement: Additional file 1: — Data abstraction form. (DOC 66 kb) [file 12879_2016_1472_MOESM1_ESM.doc]

DATA ABSTRACTION FORM

| **Record No.** | **Type Of Diagnosis** | **Date Of Diagnosis/Visit** | **Sex Of Patients (M / F )** | **status (I / O)** | **Age Of Patients** | **Place Of Residence** | **Lab confirmation** |
| --- | --- | --- | --- | --- | --- | --- | --- |
| A01 |  |  |  |  |  |  |  |
| A02 |  |  |  |  |  |  |  |
| A03 |  |  |  |  |  |  |  |
| A04 |  |  |  |  |  |  |  |
| A05 |  |  |  |  |  |  |  |
| A06 |  |  |  |  |  |  |  |
| A07 |  |  |  |  |  |  |  |
| A08 |  |  |  |  |  |  |  |
| A09 |  |  |  |  |  |  |  |
| A10 |  |  |  |  |  |  |  |
| A11 |  |  |  |  |  |  |  |
| A12 |  |  |  |  |  |  |  |
| A13 |  |  |  |  |  |  |  |
| A14 |  |  |  |  |  |  |  |
| A15 |  |  |  |  |  |  |  |
| A16 |  |  |  |  |  |  |  |
| A17 |  |  |  |  |  |  |  |
| A18 |  |  |  |  |  |  |  |
| A19 |  |  |  |  |  |  |  |
| A20 |  |  |  |  |  |  |  |
| A21 |  |  |  |  |  |  |  |
| A22 |  |  |  |  |  |  |  |
| A23 |  |  |  |  |  |  |  |
| A24 |  |  |  |  |  |  |  |
| A25 |  |  |  |  |  |  |  |
| A26 |  |  |  |  |  |  |  |
| A27 |  |  |  |  |  |  |  |
| A28 |  |  |  |  |  |  |  |
| A29 |  |  |  |  |  |  |  |
| A30 |  |  |  |  |  |  |  |
| A31 |  |  |  |  |  |  |  |
| A32 |  |  |  |  |  |  |  |
| A33 |  |  |  |  |  |  |  |
| A34 |  |  |  |  |  |  |  |
| A35 |  |  |  |  |  |  |  |
| A36 |  |  |  |  |  |  |  |
| A37 |  |  |  |  |  |  |  |
| A38 |  |  |  |  |  |  |  |
| A39 |  |  |  |  |  |  |  |
| A40 |  |  |  |  |  |  |  |

**TRENDS OF REPORTED FOODBORNE DISEASES AT THE RIDGE HOSPITAL, ACCRA: 2009- 2013**
