# Supplementary material for: Trends of reported foodborne diseases at the Ridge Hospital, Accra, Ghana: a retrospective review of routine data from 2009-2013
Source: BMC Infect Dis. 2016 Mar 24;16:139. doi: 10.1186/s12879-016-1472-8 (PMC4807551; doi:10.1186/s12879-016-1472-8)
Supplement: Additional file 3: — General Ward Register. (PDF 514 kb) [file 12879_2016_1472_MOESM3_ESM.pdf]

GENERAL WARD REGISTER

Name of Hospital: .... Name of Ward: .... Specialty: ....

Serial Number	Inpatient Number	Name of Patient	Address (Locality)	Age	Sex	Date of Admission	Provisional Diagnosis	Date of Discharge	Final Diagnosis	Additional Diagnosis	* Outcome of Admission	* Cost of Treatment	■ Insurance Scheme

* OUTCOME OF ADMISSION: Discharged, Died, Transferred, Abscond.

* COST OF TREATMENT: Indicate the full cost of hospitalisation even if patient is Exempted or Covered by Insurance.

■ INSURANCE SCHEME: Indicate the Insurance Scheme of patient, if patient has no insurance scheme indicate none.

Scanned by CamScanner
